# Different tightening schemes in thread-drawing therapy and their effects on anal function recovery in patients with high simple anal fistula

**DOI:** 10.3389/fphys.2025.1520260

**Published:** 2025-06-18

**Authors:** Pengfei Zhao, Lifang Wang, Qian Song, Shili Yuan, Dongmei Yang, Yao Liu, Tao Zhang

**Affiliations:** Department of Anorectal, Shijiazhuang TCM Hospital, Shijiazhuang, Hebei, China

**Keywords:** high simple anal fistula, precise thread-drawing therapy, influencing factors, anal function, wound symptom score, anorectal pressure, Wexner score

## Abstract

**Objective:**

We aimed to compare the effects of different tightening schemes in thread-drawing therapy on the recovery of anal function in patients with high simple anal fistulas after treatment.

**Methods:**

One hundred patients with high simple anal fistulas who met the inclusion criteria were randomly divided into four groups of 25 patients each. All patients underwent low-level incision and high-level thread-drawing surgery. In the 1/5, 1/4, 1/3, and 1/2 groups, the rubber band cutting force was applied by tightening the surrounding muscle bundle to 1/5, 1/4, 1/3, and 1/2 of its circumference, respectively (using a graduated rubber band). Subsequent tightenings were also performed to the corresponding fractions of the circumference. The overall clinical efficacy, wound healing time, wound symptom score, anal function, and Wexner score were compared among the four groups.

**Results:**

The 1/5 group had the longest wound healing time, longer than those of the 1/4, 1/3, and 1/2 groups (*p* < 0.05). On the seventh postoperative day, the 1/2 group had a higher wound symptom score than the 1/5, 1/4, and 1/3 groups (*p* < 0.05). Three months after surgery, patients in the 1/5 group had higher resting anal canal pressure and maximum anal canal systolic pressure than the other three groups; the 1/4 and 1/3 groups had higher values than the 1/2 group (*p* < 0.05). One month and 3 months after surgery, patients in the 1/2 group had the highest Wexner scores, higher than those in the 1/5, 1/4, and 1/3 groups (*p* < 0.05).

**Conclusion:**

Tightening schemes of the 1/4 and 1/3 groups were found to be optimal as they resulted in less postoperative pain and minimal impact on anal function.

## Introduction

Anal fistula is a common condition with a high incidence rate (An et al., 2023). The risk of recurrence is associated with several factors, including the following: 1) anatomical characteristics of the fistula and the existence of comorbidities, 2) inadequate preoperative evaluation, 3) intraoperative oversights, and 4) insufficient postoperative nursing in the early and late postoperative periods (Bakhtawar and Usman, 2019). Although novel classification systems have been proposed, fistulas are still commonly classified as either ‘simple’ or ‘complex’ based on their anatomical relationship to the external anal sphincter (Emile et al., 2021). A high anal fistula refers to a granulomatous tract that connects the anal canal or rectum to the perianal skin, consisting of an internal opening, fistulous tract, and external opening. In high anal fistulas, the tract runs above the levator ani muscle and the anorectal ring (Zheng et al., 2020). Since the fistulous tract involves the critical functional areas of the anal sphincter complex, the classification of the ‘high’ type suggests that the integrity of the sphincter is under significant threat during the treatment process (Chen et al., 2024). Therefore, preserving sphincter function is a key challenge for patients in this category. A high simple anal fistula, a subtype of high anal fistula, is distinguished from the high complex variant by the presence of a single fistulous tract and a single external opening (Bhat et al., 2023; Cai et al., 2023). The main therapeutic challenge lies in eradicating the fistula while preserving anal sphincter function to prevent complications such as postoperative incontinence (Bhat et al., 2023).

Sphincter-preserving fistula treatment methods have been available for decades. These methods consist of loose setons, anorectal advancement flaps, fibrin glue, collagen plugs, ligation of the intersphincteric fistula tract, and fistula laser closure (Bhat et al., 2023). Surgery is currently the most effective approach for treating anal fistulas, while medication is often used for symptomatic control. Successful outcomes rely on accurate identification and treatment of the internal opening, complete excision of the fistula tract, appropriate management of the anal sphincter, and adequate wound drainage. Seton therapy is still a reasonably valid choice because of its simplicity and optimal outcomes in the treatment of high anal fistulas (Mukherjee et al., 2022). However, during follow-up, patients often report issues such as pain during seton tightening and the need for anesthesia (Tatli et al., 2021). Despite its clinical importance, comprehensive studies on factors affecting functional recovery following precise thread-drawing therapy are limited. Therefore, this study aims to evaluate anal functional recovery in patients with high simple anal fistulas treated with precise thread-drawing therapy in order to guide the selection of the optimal seton technique in clinical practice.

## Materials and methods

### Ethical approval

This study was approved by the Research Ethics Committee of the East Hospital of Shijiazhuang Traditional Chinese Medicine Hospital. Written informed consent was acquired from all patients prior to the utilization of their clinical data.

### Study subjects

**FIGURE 1:**
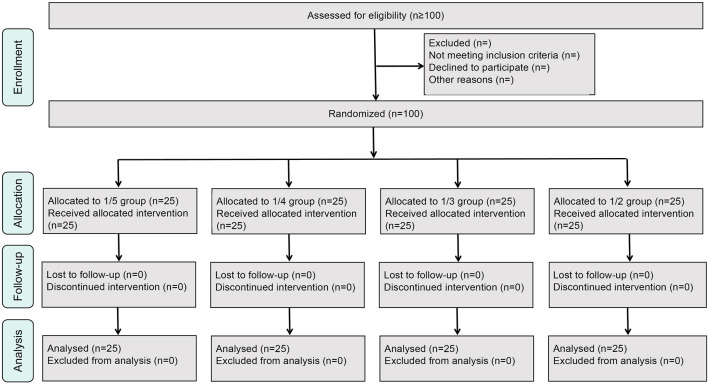
Simplified CONSORT flow diagram.

Patients hospitalized in the Anorectal Department of the East Hospital of Shijiazhuang Traditional Chinese Medicine Hospital from April 2021 to March 2023 were recruited, and 100 patients who met the diagnostic criteria for high simple anal fistulas were included in the study (a simplified CONSORT flow diagram is shown in Figure 1).

The diagnostic criteria for high simple anal fistulas were based on the “*Guidelines for Clinical Diagnosis and Treatment of Anal Fistula*” (2006 edition), jointly developed by the Anorectal Society of the China Association of Chinese Medicine, the Colorectal and Anal Surgery Group of the Surgical Branch of the Chinese Medical Association, and the Colorectal and Anal Diseases Specialized Committee of the Chinese Society of Integrative Medicine.

**FIGURE 2:**
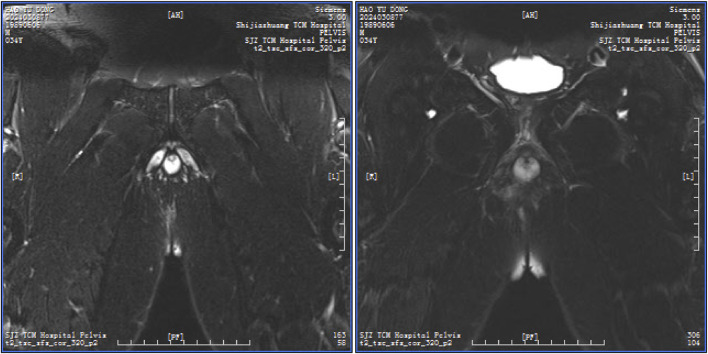
Representative imaging of high simple anal fistula patients.

A high simple anal fistula is defined as a fistula with a single tract originating from an internal opening in the anal crypt and extending above the deep external sphincter, involving structures above the puborectalis and levator ani muscles (Figure 2 shows a representative imaging example).

Inclusion criteria: the inclusion criteria included patients diagnosed with high simple anal fistula through preoperative pelvic MRI or double-plane anal canal ultrasound and digital rectal examination; patients aged between 18 and 65 years, regardless of sex; those with normal anal morphology and function before surgery; those whose internal orifice was clarified by preoperative specialist examination and three-dimensional rectal color Doppler ultrasound; and those who voluntarily agreed to take part in the study and provided signed informed consent.

Exclusion criteria: the elusion criteria included patients with trauma-induced anal fistula; specific anal fistula caused by Crohn’s disease, ulcerative colitis, tuberculosis, or actinomycosis; patients with diabetes mellitus, vitamin C deficiency, or other metabolic disorders affecting wound healing; patients with a history of previous anal fistula surgery; patients with major comorbidities involving the cardiovascular, cerebrovascular, hepatic, renal, or hematopoietic systems; patients with allergy, malignant neoplasms, psychiatric disorders, or inflammatory bowel diseases; and pregnant or breastfeeding women.

### Grouping

**FIGURE 3:**
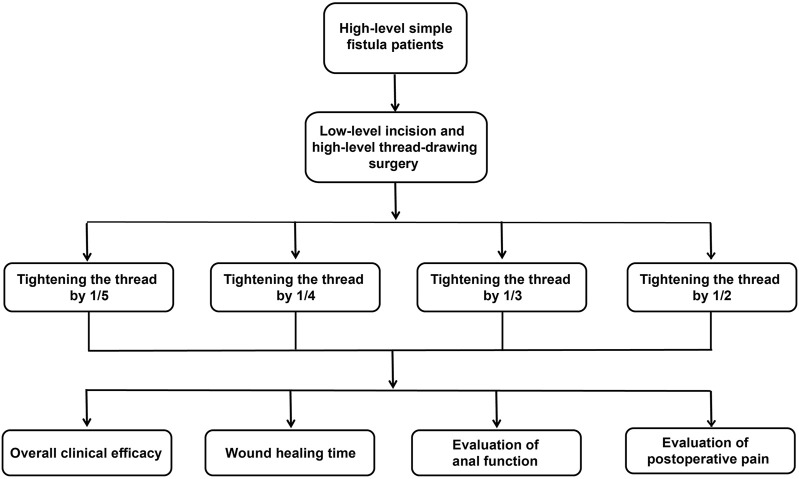
Technical route flowchart. Note: a total of 100 eligible patients with high simple anal fistulas were randomized into four groups (n = 25 per group). All patients underwent low-incision and high-seton ligation surgery: group 1/5, the seton tightening force was adjusted to constrict the enclosed muscle bundle by 1/5 of its circumference per tightening session (using a calibrated rubber band); group 1/4, the seton tightening force constricted the muscle bundle by 1/4 of its circumference per session; group 1/3, the seton tightening force constricted the muscle bundle by 1/3 of its circumference per session; group 1/2, the seton tightening force constricted the muscle bundle by 1/2 of its circumference per session. Outcome measures included the overall clinical efficacy, wound healing time, wound symptom score, anal function, and Wexner score.

One hundred eligible patients were divided into four groups according to the order of their admission date, with each group containing 25 patients, as determined by a computer-generated randomization scheme. The 1/5 group: during the operation, the rubber band was tightened by 1/5 of the circumference of the encircled muscle bundle using a scaled rubber band. Subsequent tightenings were performed every 7–8 days, each time reducing the circumference by an additional 1/5. In the 1/4, 1/3, and 1/2 groups, during the operation, the cutting force of the rubber band was tightened by 1/4, 1/3, and 1/2 of the circumference of the encircled muscle bundle, respectively. Subsequent tightenings followed the same proportional reductions (1/4, 1/3, or 1/2) at intervals of 7–8 days. A single-blind method was used, in which the outcome assessor was blinded to group allocation (Figure 3 shows the technical route flowchart).

### Treatment methods

Preoperative preparation: all patients underwent comprehensive preoperative examinations, including routine blood and urine tests, liver function, renal function, electrolytes, coagulation profile, transfusion screening, blood typing, blood sedimentation, blood glucose, tuberculosis antibody test, electrocardiogram, chest radiographs, abdominal ultrasound, electronic colonoscopy, three-dimensional rectal ultrasound, and anorectal manometry. These assessments aimed to delineate the direction of the fistula tract, identify the internal opening, and evaluate its relationship with the sphincter.

Patients fasted for 6 h before the operation. A sodium phosphate solution enema was administered the night before surgery, and the operative area was thoroughly cleaned and prepared.

**FIGURE 4:**
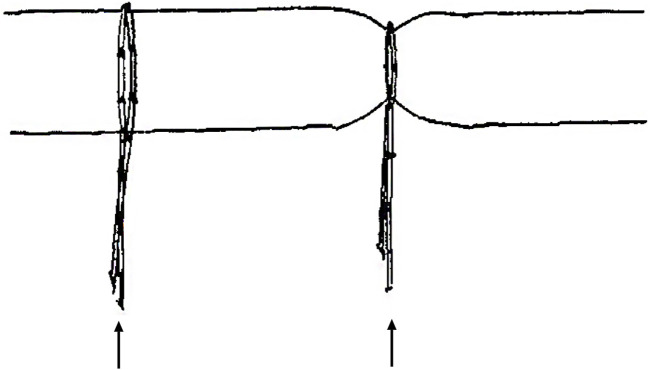
Illustration of seton placement in the 1/3 group. Note: the left rubber band, as indicated by the black arrow, is tensionless around the muscle tissue, with a circumference of 6 cm, as measured by the scale. The right rubber band, as indicated by the black arrow on the right, has been tightened by 1/3, resulting in a circumference of 6 cm × (1 − 1/3) = 4 cm around the muscle tissue.

Surgical procedures: all operations were performed by the same team of experienced colorectal surgeons. Patients in all groups underwent a standardized low-level incision and high-level thread-drawing procedure. Under sacral anesthesia and sterile conditions, a probe was used to trace the fistula tract from the external opening to the internal orifice. The fistula tract below the anal canal sphincter was first incised up to the dentate line. Necrotic tissue was excised, the tract was curetted, and adequate drainage was ensured. For the portion of the fistula above the anorectal ring, a scaled rubber band was passed through the tract and internal orifice using a probe, exiting from the incision site below the anorectal ring. The rubber band was then tightened and tied, ensuring that the seton reached the uppermost point of the fistulous cavity. The seton typically involved the muscle layer of the anorectal ring, mucosa, or a combination thereof, using a standardized double-stranded rubber band. Wound closure involved the insertion of Vaseline gauze into the anus for compression, followed by sterile gauze dressing with moderate pressure to ensure fixation and stability (Figure 4 shows a visual representation of the seton placement in the 1/3 group).

Postoperative care: patients resumed a liquid diet for 6 h after the operation and were allowed bowel movements on the second day after the operation. Oral or topical medications were provided as needed to facilitate defecation. All patients received identical postoperative care protocols. After defecation, all patients were given a sitz bath with Nuo Erkang lotion (produced by Shijiazhuang Traditional Chinese Medicine Hospital, Hebei; Registration No. Z20051133; it consists of *Dioscorea nipponica*, *Sophora flavescens*, Radix et Rhizoma Rhei, Mirabilitum, alum, *Gentiana macrophylla*, Saposhnikoviae Radix, *Zanthoxylum bungeanum*, Pollen Typhae, *Galla chinensis*, Flos Carthami, and borneolum). The sitz bath and dressing were applied twice daily, in the morning and evening. All patients were given Zigui Jiedu Ointment (produced by Shijiazhuang Traditional Chinese Medicine Hospital, Hebei; Registration No. Z20051128; it contains *Arnebia euchroma*, *Angelica sinensis*, and *Angelica dahurica*) until the wounds were healed. In the early postoperative period, wounds were irrigated with 0.5% metronidazole solution to promote fresh granulation. In the middle and late periods, the wound was irrigated with saline. Broad-spectrum antibiotics were used continuously for 3 days to prevent infection. If patients experienced severe pain, diclofenac potassium sustained-release tablets were administered orally for analgesia following the pain assessment.

### Observation indicators

Evaluation criteria for overall clinical efficacy: the clinical efficacy standard of postoperative anal fistula in this study was formulated with reference to the “*Diagnostic Efficacy Standard of Chinese Medicine Illnesses (1994 version)*” issued by the State Administration of Traditional Chinese Medicine. The criteria were as follows: cured: complete disappearance of symptoms and signs and full wound healing; significantly effective: disappearance of symptoms, improvement of signs, with ≤25% of the wound unhealed, but fresh granulation tissue present; effective: disappearance of symptoms, improvement of signs, with ≤25% of the wound unhealed, but fresh granulation tissue present; ineffective: no improvement in symptoms and signs or persistent exudation and poor wound healing with >50% of the wound unhealed and only sparse granulation tissue present. Overall effective rate = (cured + significant effective + effective cases)/total number of cases × 100%.

General postoperative conditions: these included wound healing time (days from surgery to complete wound healing), length of hospitalization (days from admission to discharge), the recurrence rate, and postoperative complications. The criteria for determining the recurrence rate and postoperative complications were as follows:

Recurrence rate: patients were followed up for 6 months after the operation through regular hospital visits or remote consultations via telephone or WeChat. Postoperative recurrence was defined as the occurrence of any of the following conditions during the follow-up period, confirmed by both clinical examination and auxiliary imaging (three-dimensional rectal ultrasound): (1) incomplete healing of the surgical incision and/or external fistula opening 6 months after surgery; (2) emergence of a new external fistula opening; and (3) local signs of recurrence such as redness, swelling, pain, rupture, or purulent discharge. The recurrence rate in each group was recorded. Additionally, postoperative complications were monitored and documented, including urinary retention on the day of surgery, wound bleeding during hospitalization, and any evidence of anal deformity detected during outpatient follow-up after discharge.

Postoperative wound symptom scoring: symptom evaluation was based on the four-tier weighted scoring method for clinical diagnosis and efficacy outlined in the *Guidelines for Clinical Research of New Chinese Medicines (2002 edition)*. Pain, exudation, edema, itching, and other wound-related symptoms were assessed on postoperative days 1, 7, 14, and 21.

Scoring of postoperative pain symptoms: pain was assessed using the visual analog scale (VAS), where 0 points indicated no pain; 1 point indicated mild pain (VAS 1–3) without the need for medication; 2 points indicated moderate pain (VAS 4–6) requiring general analgesics or rectal suppositories; and 3 points indicated severe pain (VAS 7–10) necessitating morphine analgesia. Exudation was scored as follows: 0 points for localized wetting of two or fewer dressings (6 × 6 cm, four-ply) within 12 h; 1 point for wetting of three dressings; 2 points for wetting of four or more dressings; and 3 points for profuse exudation requiring the use of cotton pads (10 × 10 cm, 3 cm thick). Edema of the wound margin was scored as follows: 0 points for no edema; 1 point for mild swelling with visible dermatoglyphics; 2 points for moderate swelling with faint dermatoglyphics; and 3 points for pronounced swelling with loss of dermatoglyphics and a shiny skin appearance. Perianal itching was scored as follows: 0 points for no itching; 1 point for occasional itching; 2 points for intermittent itching; and 3 points for persistent itching.

Changes in anorectal pressure: anorectal manometry was employed as an objective measure of anal function. Anal resting pressure (ARP) and anal maximal contraction pressure (AMCP) were selected as the primary indices for functional assessment. Measurements were performed preoperatively and at 3 months postoperatively using the Polygram 98 multi-channel gastrointestinal manometry system (Medtronic, USA) with an eight-channel water-perfused catheter.

Wexner score: the Wexner incontinence score, a subjective index of anal continence, was assessed preoperatively and at 1, 3, and 6 months postoperatively. Patients self-reported their ability to control solid stool, liquid stool, and gas; their need for protective liners; the impact on their lifestyle; and the frequency of these occurrences. Scoring criteria were as follows: 0 for never; 1 for rarely (less than once per month); 2 for sometimes (more than once per month but less than once per week); 3 for often (more than once per week but less than once per day); and 4 for always (more than once per day). The total score ranged from 0 to 20, with lower scores indicating better anal function.

### Statistical analysis

All data were processed using SPSS 26.0 (IBM SPSS Statistics, Chicago, IL, United States) and GraphPad Prism 8.0 (GraphPad software, Inc., La Jolla, CA, United States). Measurement data were presented as the mean ± standard deviation. Measurement data were compared using paired t-tests for within-group comparisons, one-way ANOVA and Tukey’s test were performed for multiple comparisons if they met normal distribution and homogeneity of variance, the Kruskal–Wallis H-test was performed for between-group comparisons, and Wilcoxon’s rank-sum test was performed for within-group comparisons if they did not meet normal distribution and homogeneity of variance. Categorical data were expressed as n (%), and groups were compared using the χ^2^ test. *P* < 0.05 was considered statistically significant.

## Results

### General information

Comparison of general information such as gender, age, course of disease, history of smoking, history of drinking, and history of diabetes among patients in the 1/5, 1/4, 1/3, and 1/2 groups showed no significant differences, indicating comparability among the groups (*p* > 0.05) (Table 1).

**TABLE 1:** Comparison of the general data among the four groups of patients [n (%), mean ± SD].

Group	Male/female individuals	Age (years)	Course of disease (year)	Body mass index	Smoking	Drinking	Diabetes
1/5 group	19/6	37.24 ± 9.01	4.48 ± 1.36	24.14 ± 2.62	13 (52.00)	10 (40.00)	4 (16.00)
1/4 group	17/8	35.60 ± 10.20	4.44 ± 1.45	23.87 ± 2.80	15 (60.00)	12 (48.00)	6 (24.00)
1/3 group	21/4	34.44 ± 11.42	4.52 ± 1.19	23.64 ± 2.69	12 (48.00)	11 (44.00)	7 (28.00)
1/2 group	16/9	37.00 ± 9.38	4.32 ± 1.49	24.07 ± 2.75	14 (56.00)	11 (44.00)	6 (24.00)
*P*-value	0.393	0.738	0.961	0.916	0.848	0.955	0.784

Note: each group consists of 25 cases.

### Clinical outcomes after treatment

The total effective rates of the 1/5 group, 1/4 group, 1/3 group, and 1/2 group were 100%, 100%, 96.00%, and 92.00%, respectively. No notable difference was witnessed when comparing these rates among the four groups (*p* > 0.05) (Table 2).

**TABLE 2:** Comparison of clinical outcomes after treatment among four groups of patients [n (%)].

Group	Cured	Significant effective	Effective	Ineffective	Total effective rate	*P*-value
1/5 group	24	0	1	0	25 (100.00%)	0.286
1/4 group	24	1	0	0	25 (100.00%)	
1/3 group	21	1	2	1	24 (96.00%)	
1/2 group	19	2	2	2	23 (92.00%)	

Note: each group consists of 25 cases.

### General postoperative conditions

There was no significant difference among the four groups in terms of hospitalization time and recurrence rates (*p* > 0.05). Postoperatively, no complications occurred in the 1/5 group. One case of wound bleeding occurred in the 1/4 group, one case of urinary retention occurred in the 1/3 group, and two cases of wound bleeding and one case of urinary retention occurred in the 1/2 group. However, there was no difference in the occurrence of complications among the four groups (*p* > 0.05). No cases of anal deformity were observed in any group. Wound healing time among the four groups was compared, and it presented a notable difference (*p* < 0.05), in which the healing time of the 1/5 group was the longest (Table 3).

**TABLE 3:** Comparison of general postoperative conditions in the four groups [n (%), mean ± SD].

Group	Wound healing time (d)	Length of hospitalization(d)	Recurrence rate	Occurrence of postoperative complications
1/5 group	37.48 ± 1.50	18.40 ± 2.22	0	0
1/4 group	34.20 ± 1.26^a^	18.08 ± 1.96	0	1 (4.00)
1/3 group	30.52 ± 1.23^ab^	17.88 ± 2.11	1 (4.00)	1 (4.00)
1/2 group	28.32 ± 1.28^abc^	17.68 ± 1.97	2 (8.00)	3 (8.00)
*P*-value	<0.001	0.648	0.286	0.262

Note: each group consists of 25 cases. a, *p* < 0.05 vs the 1/5 group; b, *p* < 0.05 vs the 1/4 group; c, *p* < 0.05 vs the 1/3 group.

### Postoperative wound symptom scores

Postoperative wound symptom scores were compared among the four groups. The results revealed that on the first postoperative day, the differences in the scores of postoperative pain symptoms, wound exudation, edema of wound margin, and perianal itching were compared among the four groups of patients, and the differences were not statistically significant (*p* > 0.05). The scores of postoperative pain symptoms, wound exudation, edema of wound margin, and perianal itching decreased in all four groups on the 7th, 14th, and 21st postoperative days compared to those on the 1st postoperative day; on the seventh postoperative day, the difference between the wound symptom scores of the 1/5 group, 1/4, and 1/3 groups was statistically significant compared with that of the 1/2 group (*p* < 0.05), but no difference was witnessed between the 1/5, 1/4, and 1/3 groups (> 0.05). On the 14th and 21st postoperative days, no differences were observed in wound symptom scores among the four groups of patients (*p* > 0.05) (Table 4).

**TABLE 4:** Comparison of postoperative wound healing and wound symptom scores among the four groups of patients (mean ± SD).

Indicator	Time	1/5 group	1/4 group	1/3 group	1/2 group
Scoring of postoperative pain symptoms (point)	On the 1st day after surgery	1.92 ± 0.64	2.04 ± 0.68	2.08 ± 0.57	2.36 ± 0.49
	On the 7th day after surgery	1.48 ± 0.51^ab^	1.52 ± 0.71^ab^	1.56 ± 0.58^ab^	2.04 ± 0.68^a^
	On the 14th day after surgery	1.32 ± 0.56^a^	1.40 ± 0.58^a^	1.52 ± 0.51^a^	1.60 ± 0.58^a^
	On the 21st day after surgery	0.88 ± 0.44^a^	0.96 ± 0.54^a^	1.04 ± 0.54^a^	1.08 ± 0.49^a^
Scoring of postoperative wound exudation (point)	On the 1st day after surgery	2.32 ± 0.63	2.48 ± 0.59	2.52 ± 0.51	2.72 ± 0.46
	On the 7th day after surgery	1.88 ± 0.53^ab^	1.96 ± 0.68^ab^	2.00 ± 0.58^ab^	2.44 ± 0.51^a^
	On the 14th day after surgery	1.36 ± 0.49^a^	1.44 ± 0.51^a^	1.48 ± 0.51^a^	1.56 ± 0.51^a^
	On the 21st day after surgery	0.60 ± 0.50^a^	0.68 ± 0.48^a^	0.76 ± 0.60^a^	0.88 ± 0.60^a^
Scoring of postoperative edema of wound margin (point)	On the 1st day after surgery	2.28 ± 0.46	2.32 ± 0.48	2.40 ± 0.50	2.60 ± 0.50
	On the 7th day after surgery	1.52 ± 0.51^ab^	1.64 ± 0.49^ab^	1.80 ± 0.58^ab^	2.32 ± 0.48^a^
	On the 14th day after surgery	0.92 ± 0.28^a^	0.96 ± 0.35^a^	1.12 ± 0.53^a^	1.247 ± 0.52^a^
	On the 21st day after surgery	0.48 ± 0.51^a^	0.56 ± 0.58^a^	0.64 ± 0.49^a^	0.76 ± 0.44^a^
Scoring of postoperative perianal itching (point)	On the 1st day after surgery	2.00 ± 0.29	2.04 ± 0.35	2.08 ± 0.28	2.24 ± 0.44
	On the 7th day after surgery	1.44 ± 0.51^ab^	1.52 ± 0.51^ab^	1.60 ± 0.50^ab^	2.00 ± 0.29^a^
	On the 14th day after surgery	0.92 ± 0.28^a^	1.04 ± 0.35^a^	1.12 ± 0.33^a^	1.16 ± 0.47^a^
	On the 21st day after surgery	0.52 ± 0.51^a^	0.64 ± 0.49^a^	0.80 ± 0.41^a^	0.84 ± 0.37^a^

Note: each group consists of 25 cases. a: *p* < 0.05 vs on the first day after surgery in the same group; b: *p* < 0.05 vs the 1/2 group.

### Anorectal pressure changes

Before surgery, the differences in the levels of ARP and AMCP among the four groups of patients were not statistically significant (*p* > 0.05), and 3 months after surgery, the levels of ARP and AMCP in each group decreased significantly compared with those before surgery (*p* < 0.05). However, ARP and AMCP levels of patients in the 1/5 group were notably higher those that of the other three groups, and those of the 1/4 and 1/3 groups were higher than that of the 1/2 group (*p* < 0.05). The difference between the 1/4 and 1/3 groups was not significant (*p* > 0.05) (Table 5).

**TABLE 5:** Comparison of anorectal pressure changes before and after surgery among the four groups of patients (mean ± SD).

Group	ARP (kPa)		AMCP (kPa)	
	Before surgery	Three months after surgery	Before surgery	Three months after surgery
1/5 group	13.21 ± 1.45	11.20 ± 1.04^a^	17.27 ± 1.81	14.79 ± 1.32^a^
1/4 group	13.15 ± 1.40	9.73 ± 0.98^abc^	17.35 ± 1.86	13.50 ± 1.23^abc^
1/3 group	14.12 ± 1.32	9.35 ± 0.90^abc^	16.97 ± 1.80	13.36 ± 1.10^abc^
1/2 group	13.56 ± 1.39	8.12 ± 0.88^ab^	17.13 ± 1.79	11.34 ± 1.02^ab^

Note: each group consists of 25 cases. a, *p* < 0.05 vs before surgery; b, *p* < 0.05 vs the 1/5 group; c, *p* < 0.05 vs the 1/2 group.

### Wexner scores

The highest preoperative and postoperative Wexner scores in the four groups of patients were 1 point, reflecting occasional gas incontinence or infrequent use of sanitary pads in a small number of patients. No difference was found in preoperative Wexner scores of the anal function of the patients in each group (*p* > 0.05). At 1 and 3 months postoperatively, the Wexner scores of patients in all groups were higher than those before surgery (*p* < 0.05), with the lowest score in the 1/5 group and the highest in the 1/2 group, and the scores in the 1/5, 1/4, and 1/3 groups were lower than that in the 1/2 group (*p* < 0.05). No differences were observed in pairwise comparisons between the 1/5, 1/4, and 1/3 groups (*p* > 0.05). At 6 months post-operation, there was no difference in Wexner scores across all groups (*p* > 0.05) (Table 6).

**TABLE 6:** Comparison of anal Wexner scores among the four groups of patients (mean ± SD).

Time	1/5 group	1/4 group	1/3 group	1/2 group
Before surgery	1.35 ± 0.58	1.29 ± 0.46	1.38 ± 0.49	1.33 ± 0.56
One month after surgery	2.75 ± 0.54^ab^	2.83 ± 0.62^ab^	2.96 ± 0.65^ab^	3.50 ± 0.77^a^
Three months after surgery	1.71 ± 0.46^ab^	1.83 ± 0.71^ab^	2.04 ± 0.45^ab^	2.54 ± 0.58^a^
Six months after surgery	0.83 ± 0.55^a^	0.92 ± 0.57^a^	1.13 ± 0.62	1.21 ± 0.41

Note: each group consists of 25 cases. a, *p* < 0.05 vs before surgery; b, *p* < 0.05 vs the 1/2 group.

## Discussion

Anal fistula is a common reason for proctological surgery and a condition that impairs anorectal function and the quality of life in patients (Garcia-Olmo et al., 2019). Seton therapy remains a rational and effective treatment option (Mukherjee et al., 2022). In this study, we aimed to compare the effects of different tightening schemes in thread-drawing therapy on the recovery of anal function in patients with high simple anal fistula after treatment.

The ligation of the intersphincteric fistula tract (Schouten et al., 2013), anal fistula plug (Jayne et al., 2019), and thread-drawing surgery (Zhang et al., 2023) are treatment methods for high anal fistulas that do not involve sphincterotomy, and they have exhibited promising clinical outcomes in the treatment of high anal fistulas. Among these methods, thread-drawing surgery, after years of development, evolution, and continuous exploration and improvement by medical professionals through generations, has given rise to various modified techniques. However, all these modifications generally fall within the category of thread-drawing surgery. The traditional cutting seton method possesses a high recurrence rate and can cause severe damage to the anal sphincter, leading to anal incontinence. Loose combined with cutting seton is a new approach developed based on the traditional technique, which has been adopted by many clinicians in China (Cheng et al., 2021). As previously reported, cutting seton for high anal fistula reaches 98% healing, and most patients have good continence, especially male individuals, with high patient satisfaction (Patton et al., 2015). Another study has demonstrated that for simple anal fistulas, tunnel thread-drawing therapy can decrease the recovery process and improve the quality of life in patients (He et al., 2009). A modified ligation of the intersphincteric fistula tract technique effectively treats high simple anal fistulas (Cai et al., 2023). Yet, the traditional cutting seton technique typically requires multiple tightening sessions, each of which can cause significant pain due to the dense innervation of the anal region, thus limiting its broader clinical application. Moreover, the large postoperative scar and local tissue defects in the anal area can impair continence, particularly affecting the control of gas and fluids (Jiang et al., 2021).

Traditional seton therapy is limited by a simplistic thread design, complex operative procedures, difficulty in quantifying tension during tightening, and significant patient discomfort. To address these shortcomings, numerous technical improvements have been explored worldwide. Our study demonstrates that modified thread-drawing approaches yield favorable clinical outcomes in high simple anal fistulas, though distinct differences in postoperative anal function recovery were observed among groups. Specifically, the wound healing time in the 1/5 group was significantly longer than that in the 1/4, 1/3, and 1/2 groups. Moreover, the 1/2 group exhibited the highest wound symptom scores on postoperative day 7, indicating increased early postoperative discomfort. At the 3-month follow-up, the 1/5 group showed the best recovery in anal canal pressure, while the 1/2 group exhibited the poorest outcomes. Wexner scores at 1 and 3 months postoperatively were also highest in the 1/2 group, suggesting more pronounced short-term anal dysfunction. A comprehensive evaluation indicated that the 1/3 and 1/4 tightening schemes were superior in preserving anal sphincter function and minimizing postoperative pain. Although the 1/4 group had a slightly longer healing time than the 1/3 group, the protective effect on anal function was comparable between the two. These findings offer important clinical guidance, supporting individualized protocol selection between the 1/3 and 1/4 tightening schemes based on patient characteristics and treatment priorities.

## Limitations

This study has several limitations. The relatively small sample size and absence of *a priori* power calculation may limit the generalizability of the findings. Furthermore, the lack of long-term follow-up precludes the evaluation of late complications and sustained anal function. Several potential confounding factors inherent in clinical practice may also have influenced the outcomes, including (1) variability in fistula tract length—even within “simple” classifications—may alter the degree of sphincter involvement and seton-induced tension; (2) occult fistulous branches missed during preoperative imaging could lead to persistent inflammation and delayed wound healing; and (3) differences in perifistular fibrosis may affect both the rate of seton cutting and the elasticity recovery of the anal sphincter postoperatively.

## Conclusion

In summary, this study demonstrates that 1/3 and 1/4 thread-drawing schemes are superior treatment options for high simple anal fistulas, offering better anal function preservation and lower postoperative discomfort than other techniques. Although the 1/4 group required slightly more time for wound healing than the 1/3 group, both protocols provided comparable functional outcomes. This provides meaningful insights for personalized clinical decision-making. For elderly patients prioritizing sphincter preservation and willing to tolerate longer healing periods, the 1/4 protocol is strongly recommended. In contrast, younger patients with greater muscle tone, higher activity levels, and a desire for shorter recovery time may benefit more from the 1/3 protocol. By comparing therapeutic outcomes across different suspension and tightening modalities, this study lays a foundation for standardized and optimized treatment protocols for high simple anal fistulas, enhancing the scientific rigor and clinical effectiveness of anorectal surgery. Globally, these findings contribute quantifiable and adaptable innovations to traditional seton therapy, particularly suited for resource-limited settings, and support the broader implementation of the “sphincter-preserving” principle outlined in international treatment guidelines. Although seton therapy is less commonly used in Western clinical practice, our technical refinements—such as staged tightening and precise tension control—offer valuable insights for enhancing existing protocols. Although further multicenter studies are needed to validate these techniques across diverse populations and healthcare systems, the core concept of function-oriented tension management provides a meaningful direction for future strategies in fistula treatment. Expanding the sample sizes and refining technical parameters in future research will further optimize treatment protocols and help deliver more precise and effective care to patients with high simple anal fistulas, ultimately accelerating their recovery and improving their quality of life.

## Data Availability

The original contributions presented in the study are included in the article/supplementary material; further inquiries can be directed to the corresponding author.
